# WTIP upregulates FOXO3a and induces apoptosis through PUMA in acute myeloid leukemia

**DOI:** 10.1038/s41419-021-04467-0

**Published:** 2021-12-20

**Authors:** Yunqi Zhu, Xiangmin Tong, Ying Wang, Xiaoya Lu

**Affiliations:** 1 PET Center, Department of Nuclear Medicine, The first Affiliated Hospital, Zhejiang University School of Medicine, Hangzhou, Zhejiang 310003 China; 2 Phase I Clinical Research Center, Zhejiang Provincial People’s Hospital, People’s Hospital of Hangzhou Medical College, Hangzhou, Zhejiang 310014 China; 3 Department of Clinical Laboratory, Zhejiang Provincial People’s Hospital, People’s Hospital of Hangzhou Medical College, Hangzhou, Zhejiang 310014 China; 4 Key Laboratory of Tumor Molecular Diagnosis and Individualized Medicine of Zhejiang Province, Zhejiang Provincial People’s Hospital, People’s Hospital of Hangzhou Medical College, Hangzhou, Zhejiang 310014 China

**Keywords:** Haematological cancer, Haematological cancer

## Abstract

Acute myeloid leukemia (AML) is an aggressive and heterogeneous clonal hematologic malignancy for which novel therapeutic targets and strategies are required. Emerging evidence suggests that WTIP is a candidate tumor suppressor. However, the molecular mechanisms of WTIP in leukemogenesis have not been explored. Here, we report that WTIP expression is significantly reduced both in AML cell lines and clinical specimens compared with normal controls, and low levels of WTIP correlate with decreased overall survival in AML patients. Overexpression of WTIP inhibits cell proliferation and induces apoptosis both in vitro and in vivo. Mechanistic studies reveal that the apoptotic function of WTIP is mediated by upregulation and nuclear translocation of FOXO3a, a member of Forkhead box O (FOXO) transcription factors involved in tumor suppression. We further demonstrate that WTIP interacts with FOXO3a and transcriptionally activates FOXO3a. Upon transcriptional activation of FOXO3a, its downstream target PUMA is increased, leading to activation of the intrinsic apoptotic pathway. Collectively, our results suggest that WTIP is a tumor suppressor and a potential target for therapeutic intervention in AML.

## Introduction

Acute myeloid leukemia (AML) is a highly heterogeneous clonal hematologic disease characterized by genetic and epigenetic alterations leading to the proliferation and expansion of abnormal myeloid stem/precursor cells in blood and bone marrow [1]. AML is the most common adult leukemia, which accounts for about one-third of all hematological malignancies [1, 2]. Although there have been significant improvements in targeted drugs, allogeneic hematopoietic stem cell transplantation, and immunotherapy for the treatment of AML [3–5], the relapse rate remains high and the 5-year overall survival is only 24% [6]. Therefore, a better understanding of the molecular mechanisms and identification of new actionable targets for the therapy of AML is crucial.

Forkhead box O (FOXO) proteins, including FOXO1, FOXO3a, FOXO4, and FOXO6, are a group of transcriptional factors that can modulate the expression of genes involved in apoptosis, cell cycle arrest, DNA repair, oxidative stress, and other cellular functions [7–9]. Increasing evidence suggests that FOXO proteins are involved in AML development and progression, including leukemogenesis, relapse, and drug sensitivity [10]. In particular, FOXO3a, the only constantly expressed FOXO protein in primary AML cells, is inactivated due to its phosphorylation and translocation from the nucleus into the cytoplasm [11]. In healthy subjects, FOXO3a is localized both in cytoplasmic and nuclear compartments, whereas it is exclusively localized within the cytoplasm of adult AML cells, suggesting its complete loss of transcriptional activity [12]. Higher levels of phosphorylated FOXO3a are an adverse prognostic factor in AML patients [13]. These results suggest that FOXO3a is a tumor suppressor in AML. However, the precise mechanisms underlying *FOXO3a* gene regulation remain elusive.

The 19q13.11 microdeletion syndrome is a clinically recognizable condition characterized by growth deficiency, microcephaly, ectodermal anomalies, and intellectual disability, suggesting missing of potentially key genes in this region [14–17]. The *WTIP* is a candidate gene that maps to chromosome 19q13.11 [15, 17]. As a member of mammalian P-body associated protein, WTIP is involved in miRNA-mediated gene silencing in human osteosarcoma cells [18]. In cervical cancer cells, downregulation of WTIP abolished BRCA2-mediated centrosome localization and resulted in abnormal cell division, suggesting that WTIP might be involved in the development of cervical cancer [19]. Recently, it was demonstrated that WTIP protein expression is significantly reduced in non-small-cell lung cancer (NSCLC) cells, and WTIP downregulation is associated with poor prognosis in NSCLC patients [20]. In our previous study, we identified a novel fusion gene named *UBA2-WTIP* in AML and found that it abrogates WTIP-mediated P-body formation [21]. While overexpression of UBA2-WTIP promotes cell proliferation, overexpression of WTIP suppresses cell proliferation in human leukemic KG1a cells [21]. These findings support the notion that WTIP is a candidate tumor suppressor. However, the molecular mechanisms of WTIP in leukemogenesis have not been explored.

In this study, we revealed that WTIP expression is downregulated and significantly associated with poor prognosis in AML patients. Overexpression of WTIP inhibited cell proliferation and colony formation in AML cells. We further showed that WTIP overexpression was effective to induce apoptosis by upregulating FOXO3a and promoting its nuclear localization. Furthermore, we found that WTIP interacts with FOXO3a and transcriptionally activates FOXO3a mRNA expression. Upon transcriptional activation of FOXO3a, its downstream target p53 upregulated modulator of apoptosis (PUMA) is increased, leading to activation of the intrinsic apoptotic pathway. These results suggest that WTIP is a tumor suppressor and a potential target for therapeutic intervention in AML.

## Materials and methods

### Clinical samples and cell lines

Bone marrow samples were obtained from primary AML patients and healthy donors with informed consent in accordance with the Declaration of Helsinki. Studies were approved by the ethics committee of Zhejiang Provincial People’s Hospital, People’s Hospital of Hangzhou Medical College. KG1a, Kasumi-1, HL-60, MOLM-13, THP-1, U937 cells (human AML cell lines), HEK293 and HEK293T (human embryonic kidney cell line) were obtained from American Type Culture Collection (ATCC, Manassas, VA). KG1a, Kasumi-1, HL-60, THP-1, and U937 cells were cultured in RPMI-1640 medium containing 10% fetal bovine serum (FBS, GIBCO, Bethesda, MD, USA). MOLM-13 cells were cultured in an IMDM medium with 10% FBS. HEK293 and HEK293T cells were cultured in a DMEM medium with 10% FBS.

### Western blot analysis

Western blot analysis was performed as previously described [22]. Antibodies used in this study are as follows: anti-PARP1 (#9532), anti-Caspase-3 (#9662), anti-Cleaved Caspase-3 (#9661), anti-Caspase-9 (#9502), anti-Cleaved Caspase-9 (#9505), anti-p53 (#9282) antibodies obtained from Cell Signaling Technology (Beverly, MA, USA), anti-Bcl-2 (ab32124), anti-Bax (ab32503), and anti-Phospho-FOXO3a(Thr32) (#9464) antibodies obtained from Abcam (Abcam, Cambridge, UK), anti-PUMA antibody (ER31215) obtained from HuaBio (Hangzhou, China), anti-WTIP antibody (PA5-48292) obtained from Thermo Fisher Scientific (Thermo Fisher Scientific, Waltham, USA), anti-FLAG antibody purchased from Sigma-Aldrich (St. Louis, MO, USA), anti-FOXO3a (#2497) and anti-GAPDH antibody (60004-1-Ig) obtained from Proteintech (Proteintech Group, Chicago, IL, USA).

### Apoptosis analysis

Cells were plated on a 6-well plate and treated with doxycycline for 48 h. For flow-cytometry-based analysis, cells were harvested and incubated for 5 min with Annexin V-FITC/PI (MultiSciences, Hangzhou, China). The samples were analyzed using Beckman Coulter Navios Flow Cytometer (Beckman, Brea, CA, USA).

### Construction of vectors

The WTIP coding sequence with a 3×FLAG-tagged sequence was amplified from HEK293 cells, and then cloned into the pLVX-tre3G vector (Clontech, CA, USA) or pCDH-MSCVMCS-EF1α-GFP+Puro (System Biosciences, Palo Alto, CA, USA) using Hieff Clone^TM^ One Step Cloning Kit (Yeasen, Shanghai, China) as previously described [21]. The FOXO3a coding sequence with a 3×FLAG-tagged sequence was amplified from HEK293 cells, and cloned into the pCDH-MSCVMCS-EF1α-GFP+Puro (System Biosciences, Palo Alto, CA, USA) using Hieff Clone^TM^ One Step Cloning Kit as previously described [23]. The sequence of shRNAs for WTIP was designed (shWTIP target sequence: 5′-CCGGCAGCGTGTGTGGACATCTCATCTCGAGATGAGATGTCCACACACGCTGTTTTTG-3′).

### Confocal microscopy analysis

Cells were fixed with 4% paraformaldehyde for 30 min, permeabilized with 0.1% Triton X-100 for 10 min and blocked with 5% BSA for 1 h, labeled with anti-WTIP and anti-FOXO3a antibodies overnight at 4 °C. After washing, cells were labeled with Alexa Fluor 488 and Alexa Fluor 594 (Yeasen, Shanghai, China) for 1 h at room temperature. Cell nuclei were stained with DAPI (Sigma-Aldrich, St. Louis, MO). Samples were analyzed using Zeiss Confocal Laser Scanning Microscope 710 (Carl Zeiss MicroImaging GmbH).

### RNA extraction, cDNA synthesis, and real-time PCR

Total RNA was isolated by TRIzol Reagent (Invitrogen, Carlsbad, CA, USA) according to the manufacturer’s instructions. cDNA was synthesized by using PrimeScript™ RT reagent Kit (TaKaRa, Dalian, China). Real-time PCR was performed by using TB Green^®^ Premix Ex Taq™ (TaKaRa). The sequences of primers for WTIP were forward, GGCATGTTACCACTGTGAGGACTG; and reverse, CGCAGGTGGCAACGACGAC. Primers for FOXO3a were forward, TGGCAAGCACAGAGTTGGATGAAG; and reverse, CATATCAGTCAGCCGTGGCAGTTC. Primers for p53 were forward, ACCGGCGCACAGAGGAAGAG; and reverse, GCCTCATTCAGCTCTCGGAACATC. Primers for PUMA were forward, CGGAGCAGCACCTGGAGTCG; and reverse, TTGAGGTCGTCCGCCATCCG. GAPDH was used as an internal control. Relative gene expression was calculated using the ΔΔCt-method.

### Co-immunoprecipitation

Cells were plated on a 6-well plate and treated with doxycycline for 48 h. Then cell lysates were prepared with RIPA lysis buffer (Beyotime, Shanghai, China). Primary antibodies or normal IgG were added to the cell lysates and incubated overnight at 4°C. The protein A magnetic beads (Bio-Rad, Hercules, CA, USA) were added to the cell lysates incubated for 2 h at 4 °C. Beads were washed three times with PBST, boiled in Laemmli buffer at 95 °C for 5 min. The supernatant was subjected to western blotting.

### Luciferase reporter assay

The promoter regions of human FOXO3a were amplified from human genomic DNA by PCR. Primers for FOXO3a promoter were forward, CTGCTCGTGGAAGGGAGGAG; and reverse, GGGGCAGCCCCCTCC. The reporters described here were derived from the pGL3-promoter luciferase vector (Promega, Madison, WI, USA). FOXO3a promoter was cloned into the pGL3-Basic vector for promoter activity analysis. HEK293T cells were grown in a 24-well plate were co-transfected with 200 ng of FOXO3a promoter vector, 20 ng of pSV-β-Galactosidase vector as an internal control, and 200 ng of WTIP full-length expression vector or control vector. The relative luciferase activity was analyzed 48 h after transfection by using the Promega E1960 Dual-Luciferase^®^ Reporter System according to the manufacturer’s instructions.

### siRNA transfection

siRNA for FOXO3a was purchased from Ribobio (Guangzhou, China). siRNA was transfected using RFect^SP^ siRNA/miRNA Transfection Reagent (Bio-trans, China) following the recommended procedures. siRNAs for FOXO3a were designed (siFOXO3a-1 target sequence: CCATGTCACACTATGGTAA; siFOXO3a-2 target sequence: GAGCTCTTGGTGGATCATC).

### Tumor xenografts mouse model

KG1a and MOLM-13 cells transfected with WTIP or control vectors were injected subcutaneously (5 × 10^6^ cells) into B-NSG mice (Beijing Biocytogen Co., Ltd). B-NSG mice were treated daily with doxycycline (2 μg/ml) by oral gavage, and tumor volumes were measured every 5 days for 20 days. Tumors were harvested on day 20 after treatment and immediately fixed in 4% paraformaldehyde. Tumor volume (V) was calculated by using the formula (L × S × S) × 0.5, where L and S were the long and short dimensions, respectively [24]. Tumor tissue paraffin sections (5 µm) were stained with anti-Ki67 antibody (#GB111141, Servicebio, Wuhan, China) for cell proliferation according to the manufacturer’s instructions. Apoptotic nuclei were visualized by using a Fluorescein In Situ Cell Death Detection Kit according to the manufacturer’s instructions (Roche Diagnostic, Mannheim, Germany). The slides were analyzed under a fluorescence microscope (Zeiss Axio Vert.A1). Animal experiments were approved by the ethics committee of Zhejiang Provincial People’s Hospital, People’s Hospital of Hangzhou Medical College.

### Statistical analysis

All data were expressed as mean ± SD and were based on experiments performed at least three times. Statistical analysis was performed using the Prism software (GraphPad 6.0). Statistical significance was calculated by Student’s *t*-test or one-way analysis of variance. *P* < 0.05 was considered to be statistically significant.

## Results

### Low levels of WTIP correlate with poor prognosis in AML patients

**Fig. 1:**
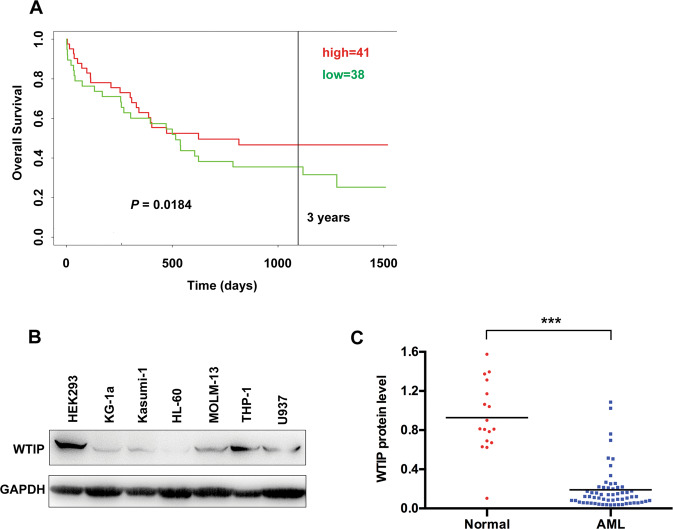
Low expression of WTIP correlates with poor prognosis in AML patients. **A** Predicting the effect of WTIP expression on the survival of AML patients using PROGgeneV2. AML patients were classified into high and low WTIP expression subgroups (as per the median). Kaplan–Meier survival curves showed that patients with low WTIP expression had significantly shorter overall survival. *P* value was calculated by log-rank test. **B** Western blot analysis of WTIP expression in the indicated AML cell lines, GAPDH served as a loading control. **C** Western blot analysis of WTIP expression in mononuclear bone marrow cells derived from 60 AML patients and 17 healthy donors. *P* values were calculated by non-paired Student’s *t*-test (***P < 0.001).

PROGgeneV2 is a web tool that can be used to study prognostic implications of genes in various cancers [25]. We initially used PROGgeneV2 database to explore the potential prognostic value of WTIP expression for clinical AML patients. We found that patients with low levels of WTIP had shorter overall survival compared with patients with high levels of WTIP (Fig. 1A). Next, we analyzed WTIP protein expression in a variety of human AML cell lines (KG1a, Kasumin-1, MOLM-13, HL-60, THP-1, and U937 cells) using western blot. AML cells expressed low levels of WTIP protein compared with HEK293 cells (Fig. 1B). To further investigate whether WTIP expression is downregulated in AML patients, we analyzed WTIP expression in a cohort of 60 primary AML patients and 17 healthy controls. Western blot results showed that WTIP levels were significantly lower in AML patients compared with healthy controls (Fig. 1C and Supplementary Fig. 1A, B). These results suggest that WTIP expression is significantly reduced both in AML cell lines and clinical specimens compared with normal controls, and low levels of WTIP are associated with poor prognosis in AML patients.

### Overexpression of WTIP inhibits cell proliferation and colony formation in AML cells

**Fig. 2:**
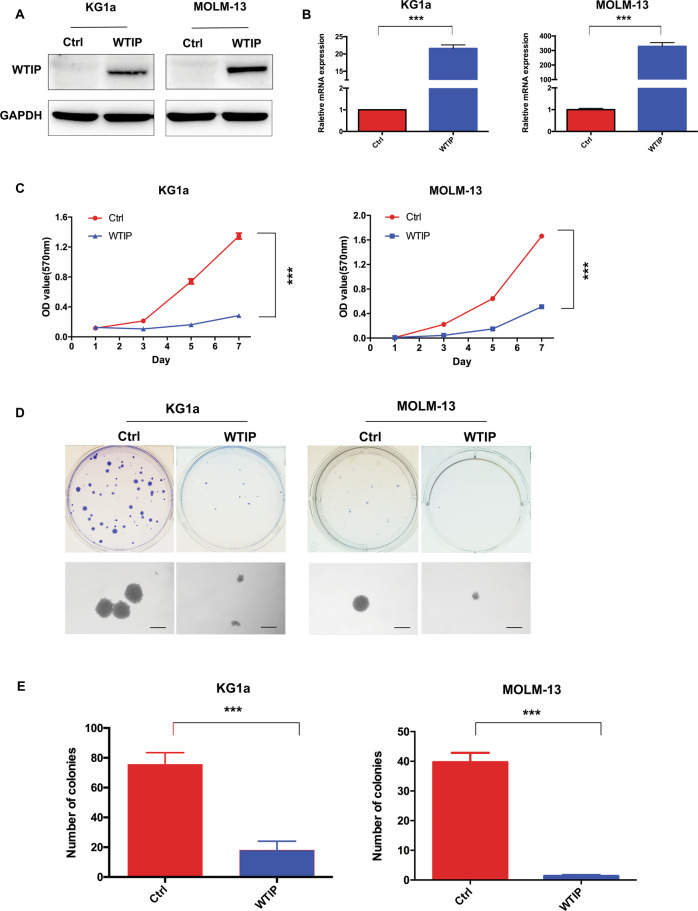
Overexpression of WTIP inhibits cell proliferation in AML cells. **A** Western blot analysis of WTIP expression in KG1a and MOLM-13 cells transfected with WTIP or control vector. **B** RT-PCR analysis of WTIP mRNA expression in KG1a and MOLM-13 cells transfected with WTIP or control vector. Data are represented as mean ± SD from three independent experiments (***P < 0.001). **C** Cell proliferation was detected by MTT assay in KG1a and MOLM-13 cells transfected with WTIP or control vector. Data are represented as mean ± SD from three independent experiments (***P < 0.001). **D** MTT assay of KG1a and MOLM-13 cells transfected with WTIP vector shows a decrease in the number of colonies compared with control vector cells. Scale bars, 50 μm. **E** Colony numbers were calculated and data are represented as mean ± SD from three independent experiments (***P < 0.001).

To further investigate the functional role of WTIP in AML, we developed a doxycycline-inducible expression system for WTIP in KG1a and MOLM-13 cells. As determined by real-time PCR and western blot analysis, in the presence of doxycycline, robust induction of WTIP at both protein and mRNA levels was observed in KG1a and MOLM-13 cells transfected with WTIP vector compared with control vector cells (Fig. 2A, B). MTT and colony formation assay were employed to examine the effects of WTIP overexpression on cell proliferation. Overexpression of WTIP in KG1a and MOLM-13 cells significantly inhibited cell proliferation compared with control vector cells (Fig. 2C). Furthermore, WTIP overexpression led to a marked decrease in the number of colonies in KG1a and MOLM-13 cells compared with control vector cells (Fig. 2D, E). These results demonstrate that overexpression of WTIP inhibits cell proliferation and leukemogenesis in AML cells in vitro.

### Overexpression of WTIP induces apoptosis by activating the intrinsic apoptotic pathway

**Fig. 3:**
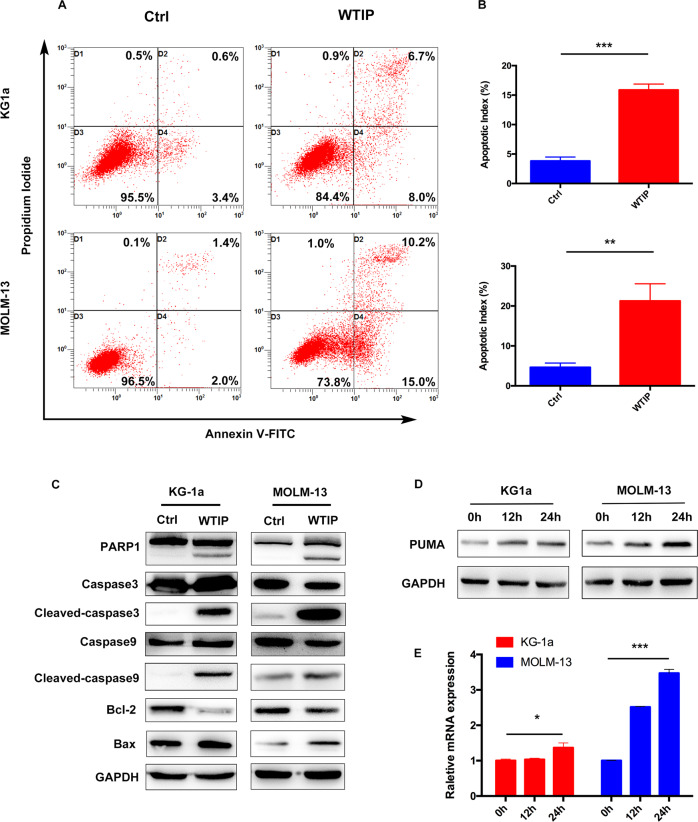
Overexpression of WTIP induces apoptosis by activating the intrinsic apoptotic pathway. **A**, **B** Flow-cytometric analysis of apoptosis in KG1a and MOLM-13 cells transfected with WTIP or control vector, as determined by using Annexin V-FITC and propidium iodide (PI). Data are represented as mean ± SD from three independent experiments (**P < 0.01). **C** Western blot analysis of apoptosis-related proteins as indicated in KG1a and MOLM-13 cells transfected with WTIP or control vector. **D** KG1a and MOLM-13 cells transfected with WTIP vector were treated with doxycycline for the indicated times, cell lysates were subjected to immunoblotting with anti-PUMA antibody. **E** KG1a and MOLM-13 cells transfected with WTIP vector were treated with doxycycline for the indicated times, total RNA was extracted and analyzed for PUMA mRNA expression using RT-PCR. Data are represented as mean ± SD from three independent experiments (*P < 0.05 and ***P < 0.001).

In order to study the molecular mechanisms by which overexpression of WTIP inhibited cell proliferation, we investigated whether apoptosis is induced. Flow cytometry results showed that Annexin V-positive and PI-positive cell population was significantly higher in WTIP-overexpressing KG1a and MOLM-13 cells compared with control vector cells (Fig. 3A, B). Western blot was employed to analyze apoptosis-related proteins in WTIP-overexpressing KG1a and MOLM-13 cells. The results showed that expression of the anti-apoptotic protein Bcl-2 was reduced, whereas the proapoptotic Bax was increased in WTIP-overexpressing KG1a and MOLM-13 cells compared with control vector cells (Fig. 3C). Moreover, we found that cleaved caspase-9, cleaved caspase-3, and cleaved PARP1 were also increased in WTIP-overexpressing KG1a and MOLM-13 cells (Fig. 3C). These results suggest that WTIP induces apoptosis by activating the intrinsic apoptotic pathway in AML cells.

The PUMA, a member of BH3-only Bcl-2 family proapoptotic protein, was identified as a critical mediator of AML cell apoptosis induced by anti-leukemic agents [26–28]. It was shown that PUMA activates Bax by inducing Bax conformational change and translocation to mitochondria in leukemic cells [29]. In the presence of doxycycline, we observed a robust induction of PUMA at both the mRNA and protein levels in KG1a and MOLM-13 cells transfected with WTIP vector (Fig. 3D, E), suggesting that PUMA is involved in WTIP overexpression induced apoptosis in AML cells.

### FOXO3a is required for WTIP-induced PUMA activation

PUMA was initially identified as a p53-inducible gene and activated in response to genotoxic stress, consequently mediating p53-dependent apoptosis [30, 31]. In addition, it was shown that PUMA is also activated by FOXO3a to initiate p53-independent apoptotic responses to nongenotoxic stimuli [30–32]. While MOLM-13 cells are p53 wild-type, KG1a cells are p53 deficient, which suggest that the regulation of PUMA by WTIP may be independent of p53 pathway. To explore this possibility, we assessed the expression of p53 protein and mRNA in MOLM-13 cells transfected with WTIP vector. The results showed that p53 protein and mRNA expression were unchanged in WTIP-overexpressing MOLM-13 cells (Supplementary Fig. 2), which further strengthens the idea that the regulation of PUMA by WTIP is independent of p53.

**Fig. 4:**
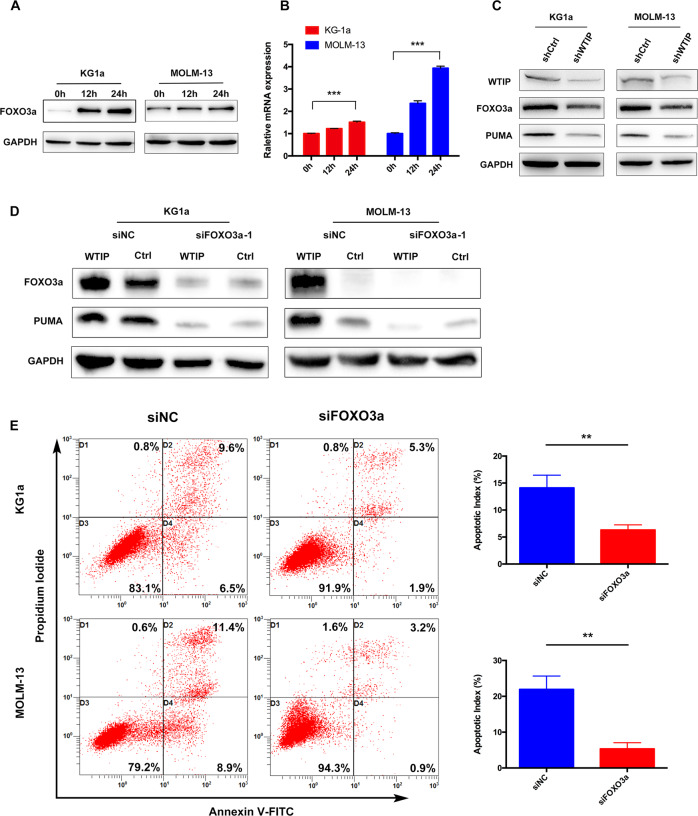
FOXO3a is required for WTIP-induced PUMA activation. **A**, **B** KG1a and MOLM-13 cells transfected with WTIP vector were treated with doxycycline for the indicated times, expression of FOXO3a protein and mRNA were detected by western blot analysis and RT-PCR, respectively. Data are represented as mean ± SD from three independent experiments (***P < 0.001). **C** Western blot analysis of the indicated proteins in KG1a and MOLM-13 cells transfected with shWTIP or shCtrl (control vector). **D** Western blot analysis of FOXO3a and PUMA expression in WTIP-overexpressing KG1a and MOLM-13 cells transfected with FOXO3a siRNAs (siFOXO3a-1) or negative control siRNA (siNC). **E** Flow-cytometric analysis of apoptosis in KG1a and MOLM-13 cells transfected with FOXO3a siRNAs (siFOXO3a-1) or negative control siRNA (siNC), as determined by using Annexin V-FITC and propidium iodide (PI). Data are represented as mean ± SD from three independent experiments (**P < 0.01).

We then sought to determine whether FOXO3a is involved in PUMA-mediated apoptosis in AML cells. In the presence of doxycycline, we observed a robust induction of FOXO3a at both the protein and mRNA levels in KG1a and MOLM-13 cells transfected with WTIP vector (Fig. 4A, B). Next, KG1a and MOLM-13 cells were transfected with WTIP shRNA lentivirus (shWTIP) to stably knock down WTIP expression. KG1a and MOLM-13 cells harboring shWTIP showed effective knockdown of WTIP at the protein level (Fig. 4C). Knockdown of WTIP downregulated FOXO3a and PUMA protein expression in KG1a and MOLM-13 cells (Fig. 4C). These results demonstrate that FOXO3a and PUMA are potential downstream targets of WTIP.

To further explore the involvement of FOXO3a in WTIP-mediated upregulation of PUMA, KG1a and MOLM-13 cells were infected with two different siRNAs (siFOXO3a-1 and siFOXO3a-2) to knock down FOXO3a expression. KG1a and MOLM-13 cells transfected with either siFOXO3a-1 or siFOXO3a-2 showed effective knockdown of FOXO3a at the mRNA level compared with cells transfected with negative control siRNA (siNC) (Supplementary Fig. 3). Western blot results showed that knockdown of FOXO3a abolished the increase of PUMA in response to WTIP overexpression in KG1a and MOLM-13 cells (Fig. 4D). Moreover, flow cytometry results showed that knockdown of FOXO3a abolished WTIP overexpression induced apoptosis (Fig. 4E). These results suggest that FOXO3a plays an essential role in WTIP overexpression induced apoptosis in AML cells.

### *FOXO3* is transcriptionally regulated by WTIP

**Fig. 5:**
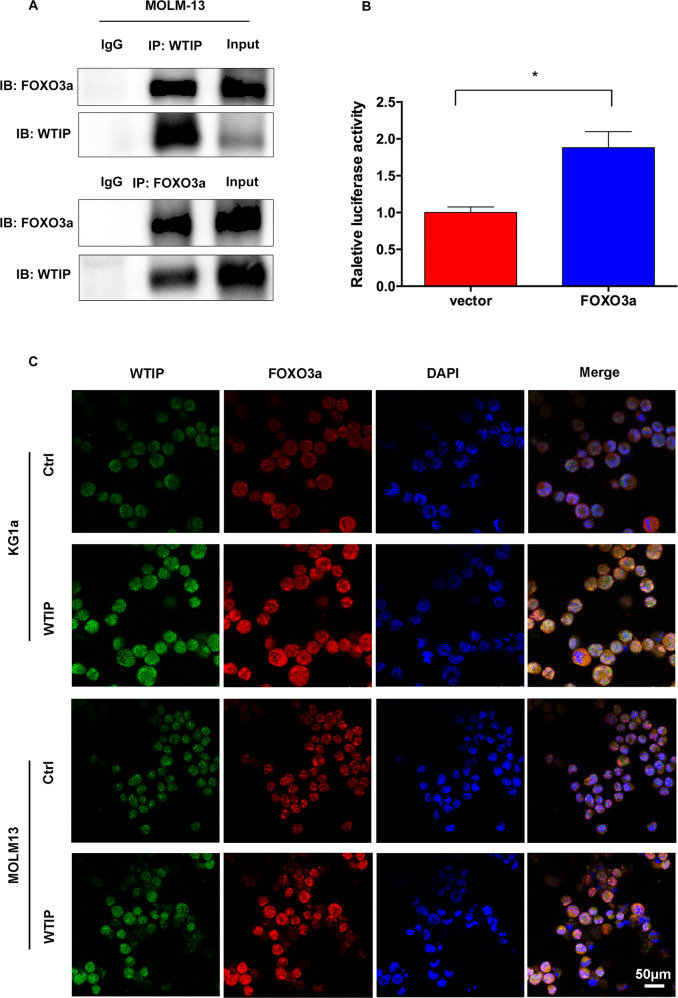
*FOXO3* gene expression is transcriptionally regulated by WTIP. **A** MOLM-13 cells transfected with WTIP or control vector were treated with doxycycline for 48 h, and cell lysates were subjected to immunoprecipitation and immunoblotting with anti-WTIP and anti-FOXO3a antibodies, respectively. **B** FOXO3a-Luc reporter vector was transfected into HEK293 cells, either in the presence of WTIP or control vector, and luciferase activity was measured 48 h after transfection. Data are represented as mean ± SD from three independent experiments (*P < 0.05). **C** Confocal immunofluorescence analysis of FOXO3a localization in KG1a and MOLM-13 cells transfected with WTIP or control vector. Scale bars, 50 μm.

The requirement of FOXO3a for WTIP-induced apoptosis implies that there might be a functional interaction between these two factors. To explore this possibility, we performed co-immunoprecipitation assay in MOLM-13 cells transfected with WTIP vector. The results revealed a physical interaction between WTIP and FOXO3a in WTIP-overexpressing MOLM-13 cells (Fig. 5A).

To determine whether WTIP can transcriptionally regulate *FOXO3a* gene expression, we generated reporter constructs in which luciferase expression is driven by a FOXO3a promotor (FOXO3a-Luc), and analyzed luciferase activity in the presence of WTIP or control vector in HEK293T cells. Co-transfection of FOXO3a-Luc reporter vector with WTIP vector increased luciferase activity in HEK293T cells compared with control vector cells (Fig. 5B). These results suggest that *FOXO3a* is transcriptionally regulated by WTIP.

As FOXO3a exerts its transcriptional activity in the nucleus, we performed a confocal immunofluorescence assay to investigate the subcellular distribution of FOXO3a in KG1a and MOLM-13 cells transfected with WTIP vector. In the absence of doxycycline, low levels of FOXO3a were observed and it is mainly localized in the cytoplasm (Fig. 5C). In the presence of doxycycline, high levels of FOXO3a were observed in both the nucleus and cytoplasm. Moreover, confocal results showed that WTIP and FOXO3a co-localized in both the nucleus and cytoplasm in KG1a and MOLM-13 cells (Fig. 5C). We then investigated FOXO3a phosphorylation on T^32^, a key regulatory site for nuclear translocation [11]. The results showed that phosphorylated FOXO3a was unchanged in WTIP-overexpressing cells compared with control vector cells (Supplementary Fig. 4). These results suggest that WTIP interacts with FOXO3a and promotes the nuclear translocation of FOXO3a in AML cells.

### WTIP inhibits tumor growth and induces apoptosis in vivo

**Fig. 6:**
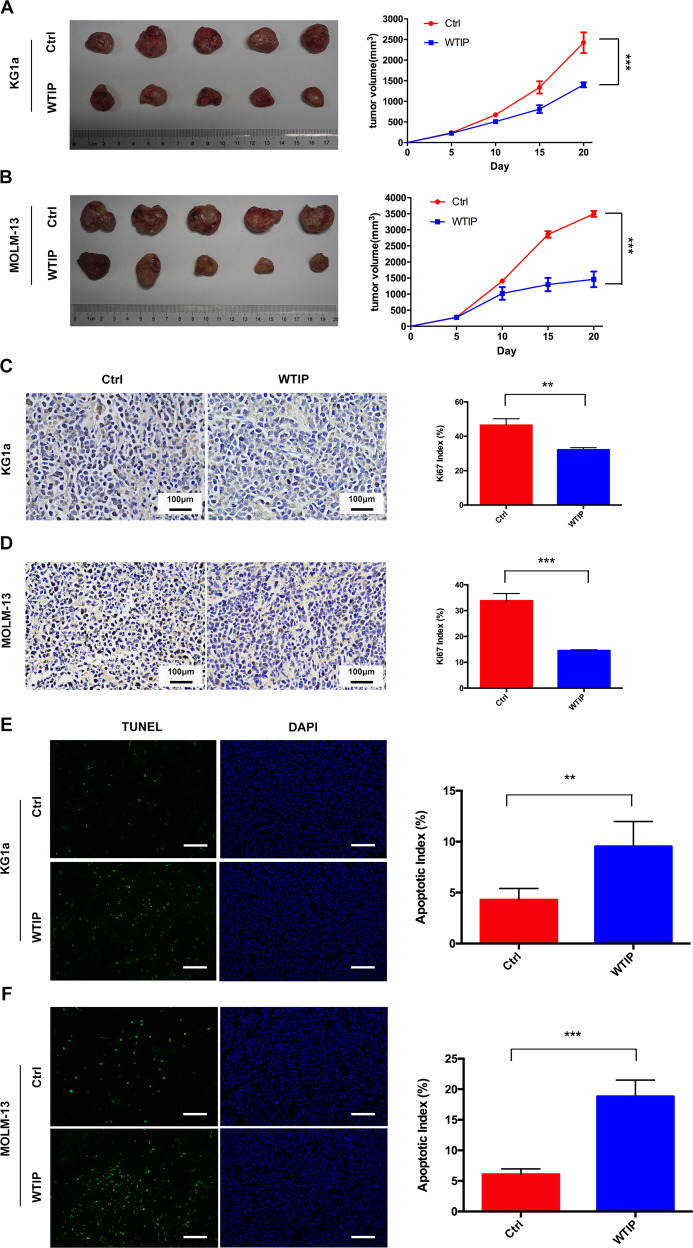
WTIP inhibits tumor growth and induces apoptosis in vivo. KG1a and MOLM-13 cells transfected with WTIP or control vector were subcutaneously injected into B-NSG mice (five mice per group). B-NSG mice were treated daily with doxycycline (2 μg/ml) by oral gavage for 20 days. **A**, **B** The picture of tumors was taken at day 20 (left) and tumor size was measured every 5 days for 20 days (right). **C**, **D** Tumor tissue sections were stained with Ki67 antibody for cell proliferation. Ki67-positive cells were calculated and represented as Ki67 proliferation index. Scale bars, 100 μm. **E**, **F** Induction of apoptosis in vivo was evaluated by TUNEL staining using histological samples at day 20. TUNEL-positive cells were calculated and represented as an apoptotic index. Scale bars, 50 μm. All data are presented as mean ± SD (**P < 0.01 and ***P < 0.001).

To explore the tumor-suppressive effects of WTIP in vivo, KG1a and MOLM-13 cells transfected with WTIP or control vectors were injected subcutaneously into B-NSG mice. The tumor volume in B-NSG mice subcutaneously injected with WTIP-overexpressing KG1a or MOLM-13 cells were significantly decreased compared with mice injected with control vector cells (Fig. 6A, B), suggesting that WTIP suppresses tumor growth in vivo. The percentage of Ki67-positive cells (the Proliferation Index, PI) was significantly decreased in WTIP-overexpressing tumors compared with control tumors (Fig. 6C, D). TUNEL staining assay showed that the apoptotic cells were significantly increased in WTIP-overexpressing tumors compared with control tumors (Fig. 6E, F). Together, these results suggest that WTIP inhibits tumor growth and induces apoptosis in vivo.

## Discussion

Over the past few decades, our understanding of the molecular pathogenesis of AML has been greatly advanced by genomics studies. Genetic abnormalities have been known to play an essential role in the pathogenesis of AML [33, 34]. Deletion of chromosome 19q13.11 is rarely reported in a variety of patients that displayed developmental delay, ectodermal dysplasia, microcephaly, intellectual disability, and genital malformations in males [14–17]. WTIP and UBA2 have been proposed to contribute to the clinical characteristics observed in these patients [15–17]. Previous studies suggested that WTIP is a candidate tumor suppressor. In cervical cancer cells, downregulation of WTIP abolished BRCA2-mediated centrosome localization and resulted in abnormal cell division, suggesting that WTIP might be involved in the development of cervical cancer [19]. A recent study reported that WTIP is downregulated in NSCLC, and low levels of WTIP are associated with poor prognosis in NSCLC patients [20]. In line with these findings, our results demonstrate that WTIP expression is significantly reduced both in AML cell lines and clinical specimens compared with normal controls, and low levels of WTIP are associated with decreased overall survival in AML patients. Moreover, overexpression of WTIP inhibited cell proliferation and colony formation by inducing apoptosis in AML cells. Our results combined with previous results suggest that loss of WTIP tumor-suppressive functions due to genetic defects may represent a common feature of tumorigenesis in various cancers.

FOXO proteins have been shown to act as tumor suppressors in several cancers [7–9]. Previous studies suggested that FOXO3a plays a crucial role in AML development and progression [10]. In healthy subjects, FOXO3a is localized both in cytoplasmic and nuclear compartments, whereas it is exclusively localized within the cytoplasm of adult AML cells, suggesting its complete loss of function [12]. AML patients with the FLT3-ITD mutation tend to have a poor prognosis compared with patients without this mutation [35, 36]. Interestingly, FLT3-ITD was shown to activate Akt and subsequently promote FOXO3a phosphorylation [37]. Phosphorylation of FOXO3a promotes its translocation from the nucleus into the cytoplasm, thereby suppressing FOXO3a-mediated apoptosis [37]. Hypomethylating agents were found to restore FOXO3 function in AML patients by increasing its expression and re-translocation to the nucleus [27, 38]. In accordance with previous reports, we found that FOXO3a is required for WTIP-induced apoptosis in AML cells. By using RT-PCR and western blot analysis, our results showed that both FOXO3a mRNA and protein levels were increased in response to WTIP overexpression. Luciferase reporter assay and confocal results further showed that WTIP interacts with FOXO3a and promotes the nuclear translocation of FOXO3a. However, we found that phosphorylation of FOXO3a was unchanged in response to WTIP overexpression. In line with this, Dey et al. found that while the mRNA and protein levels of FOXO3a were increased in response to estrogen receptor expression in prostate cancer cells, the phosphorylation of FOXO3a remained unchanged. These results suggest that overexpression of WITP activates FOXO3a transcriptional activity to produce enough unphosphorylated FOXO3a, thereby promoting its nuclear translocation [32].

PUMA is of particular interest in the regulation of AML cell apoptosis [26, 27]. In AML cells, PUMA was identified as a critical mediator of apoptosis induced by bryostatin 5, which has a significant anti-leukemic effect [26]. The anti-leukemic effects of hypomethylating agents are mediated in part by upregulation of FOXO3a and PUMA expression [27, 38]. As our results demonstrate, both mRNA and protein levels of PUMA were increased in response to WTIP overexpression. Moreover, knocked down of FOXO3a by siRNA abolished the increase of PUMA in response to WTIP overexpression. These results suggest that FOXO3a-mediated upregulation of PUMA is essential for apoptotic induction in WTIP-overexpressing AML cells. PUMA was also shown to be activated by p53-dependent stimuli [30, 31]. As the KG1a cells are p53 deficient, we investigated the involvement of p53 in WTIP-mediated apoptosis in MOLM-13 cells. We found that p53 protein and mRNA levels were unchanged in WTIP-overexpressing MOLM-13 cells, suggesting that the regulation of PUMA by WTIP is independent of p53 pathway. We also found that the expression of Bcl-2 was reduced, accompanied by activation of Bax, caspase-9, caspase-3 and PARP1 in WTIP-overexpressing cells, suggesting activation of the intrinsic apoptotic pathway. Collectively, our results demonstrate a critical role of the WTIP/FOXO3a/PUMA pathway in the regulation of cell proliferation and induction of apoptosis in AML.

We recently reported a novel fusion gene named *UBA2-WTIP* in AML and found that it abrogates WTIP-mediated P-body formation [21]. As WTIP expression is reduced both in AML cell lines and clinical specimens, we propose that UBA2-WTIP fusion resulted in the loss of function of WTIP, thus uncontrolled proliferation and expansion of abnormal cells in AML. Restoring WTIP activity may represent an attractive therapeutic strategy in the prevention and/or treatment of AML. UBA2 is a catalytic-subunit of E1-activating enzyme in the SUMOylation system [39]. Previous studies have established that *UBA2* gene mutation is associated with congenital dysplasia [40–42]. Recently, it was demonstrated that UBA2 is highly expressed in a variety of cancers, including liver cancer, NSCLC, gastric cancer, and colorectal cancer [43–47]. Therefore, it is possible that UBA2 might also play a critical role in AML cell proliferation and expansion. However, the function and clinical significance of UBA2 in leukemogenesis are largely unknown and need further investigation.

In conclusion, to our knowledge, this is the first study to elucidate the critical role of WTIP in AML. Our results demonstrate that WTIP expression is significantly reduced in AML, and low levels of WTIP are associated with decreased overall survival in AML patients. Overexpression of WTIP inhibits cell proliferation and colony formation by inducing apoptosis in AML cells. We further demonstrate that *FOXO3a* is a target gene of WTIP, and the apoptotic function of WTIP is mediated by upregulation and nuclear translocation of FOXO3a. Upon transcriptional activation of FOXO3a, its downstream target PUMA is increased, leading to activation of the intrinsic apoptotic pathway. Our results demonstrate that WTIP plays an important role in the regulation of cell proliferation and induction of apoptosis in AML. Restoring WTIP activity represents a plausible new therapeutic strategy in the treatment of AML.

## Supplementary information

Reproducibility checklist

Supplementary information

## Data Availability

The datasets generated during and/or analyzed during the current study are available from the corresponding author on reasonable request.
