# Supplementary material for: WTIP upregulates FOXO3a and induces apoptosis through PUMA in acute myeloid leukemia
Source: Cell Death Dis. 2021 Dec 20;13(1):18. doi: 10.1038/s41419-021-04467-0 (PMC8688515; doi:10.1038/s41419-021-04467-0)
Supplement: Supplementary file 2 — Supplementary information [file 41419_2021_4467_MOESM2_ESM.docx]

**Supplementary Fig. 1 Western blot analysis of WTIP expression in mononuclear bone marrow cells.** Western blot analysis of WTIP expression in mononuclear bone marrow cells derived from 60 AML patients (A) and 17 healthy donors (B). GAPDH served as a loading control.


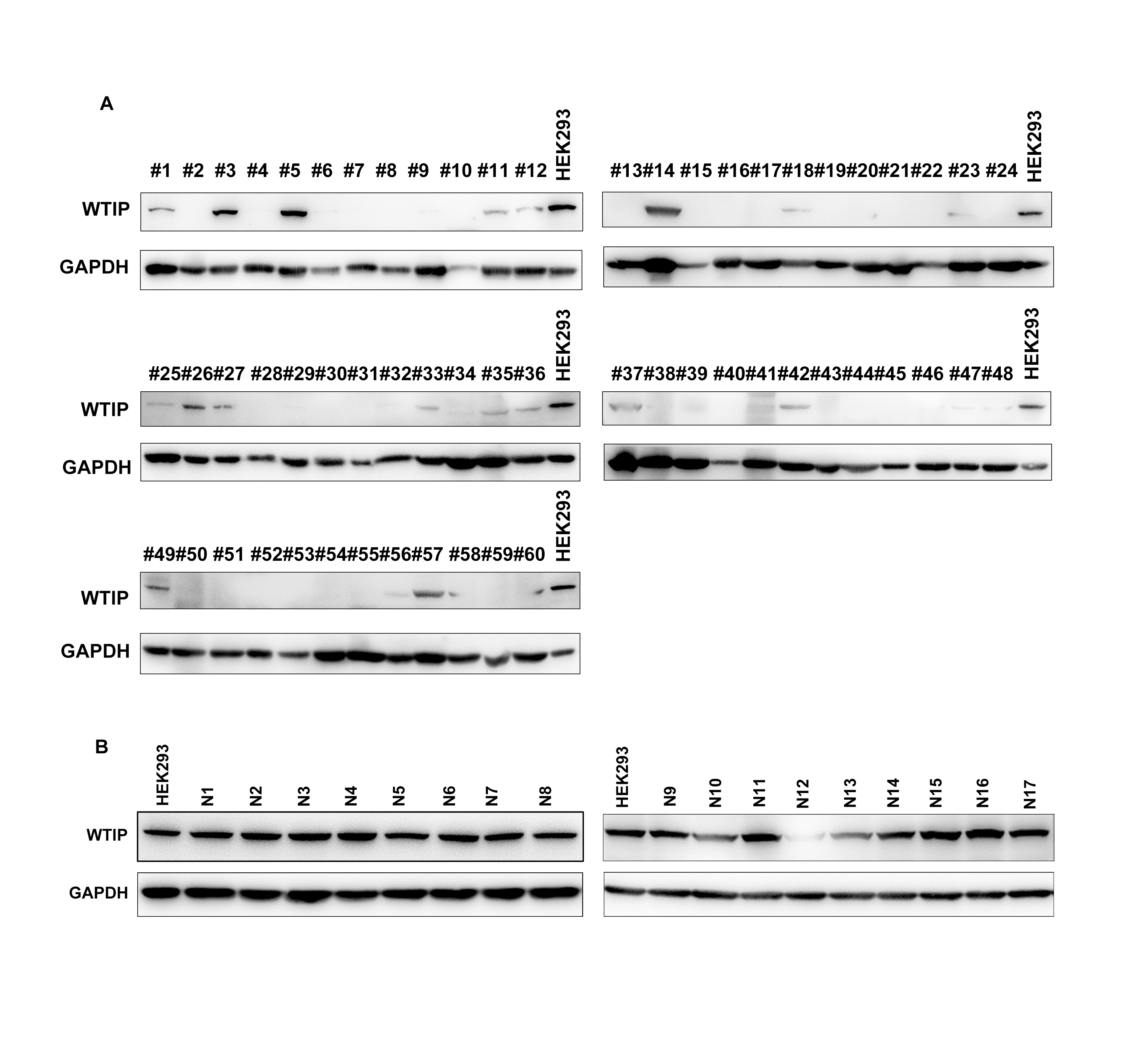


**Supplementary Fig. 2 p53 levels were unchanged in WTIP-overexpressing MOLM-13 cells.** MOLM-13 cells transfected with WTIP or control vectors were treated with doxycycline, expression of p53 protein (**A**) and mRNA (**B**) were analyzed by western blot and RT-PCR, respectively.

**
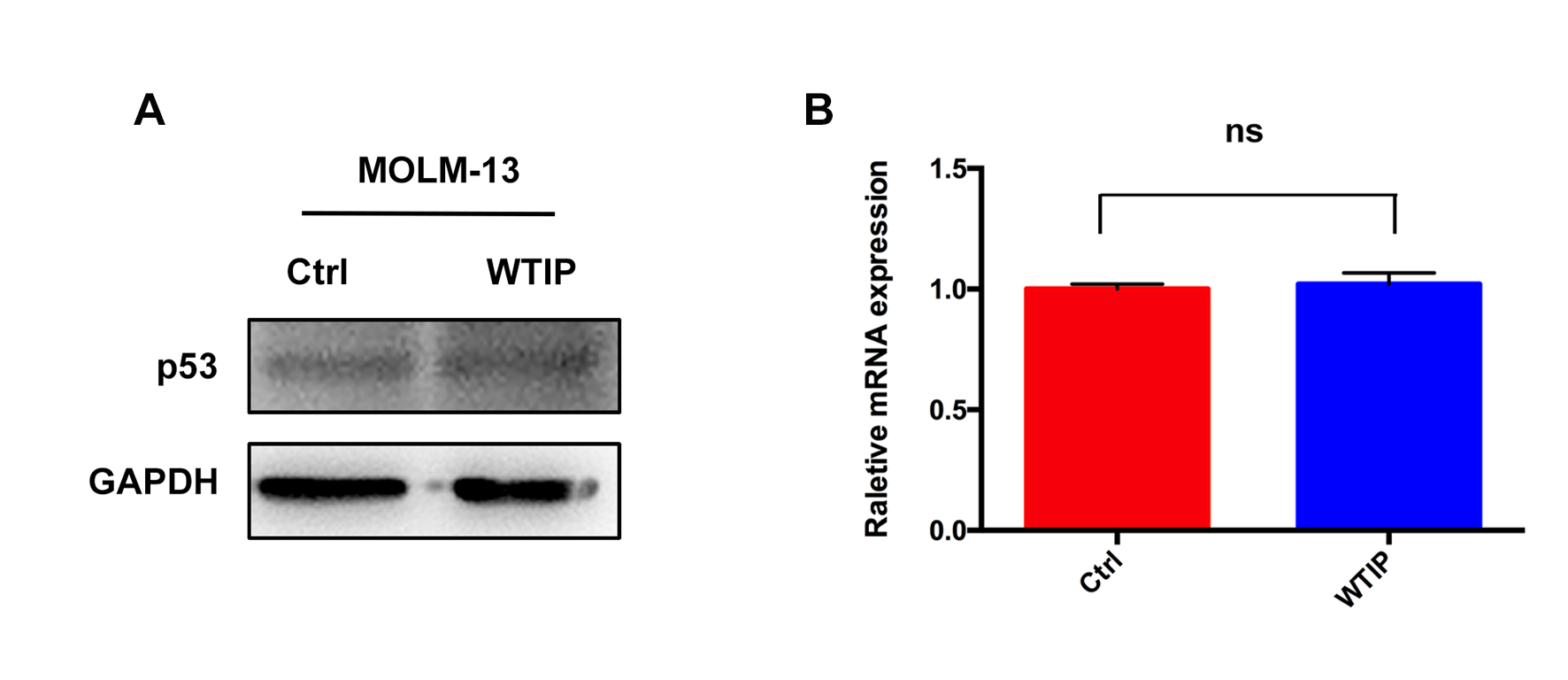
**

**Supplementary Fig. 3 Knockdown of FOXO3a in KG1a and MOLM-13 cells.** RT-PCR analysis of FOXO3a mRNA expression in KG1a and MOLM-13 cells transfected with two different siRNAs (siFOXO3a-1 and siFOXO3a-2) or negative control siRNA (siNC). Data are represented as mean ± SD from three independent experiments (***P* < 0.01 and ****P* < 0.001).

**
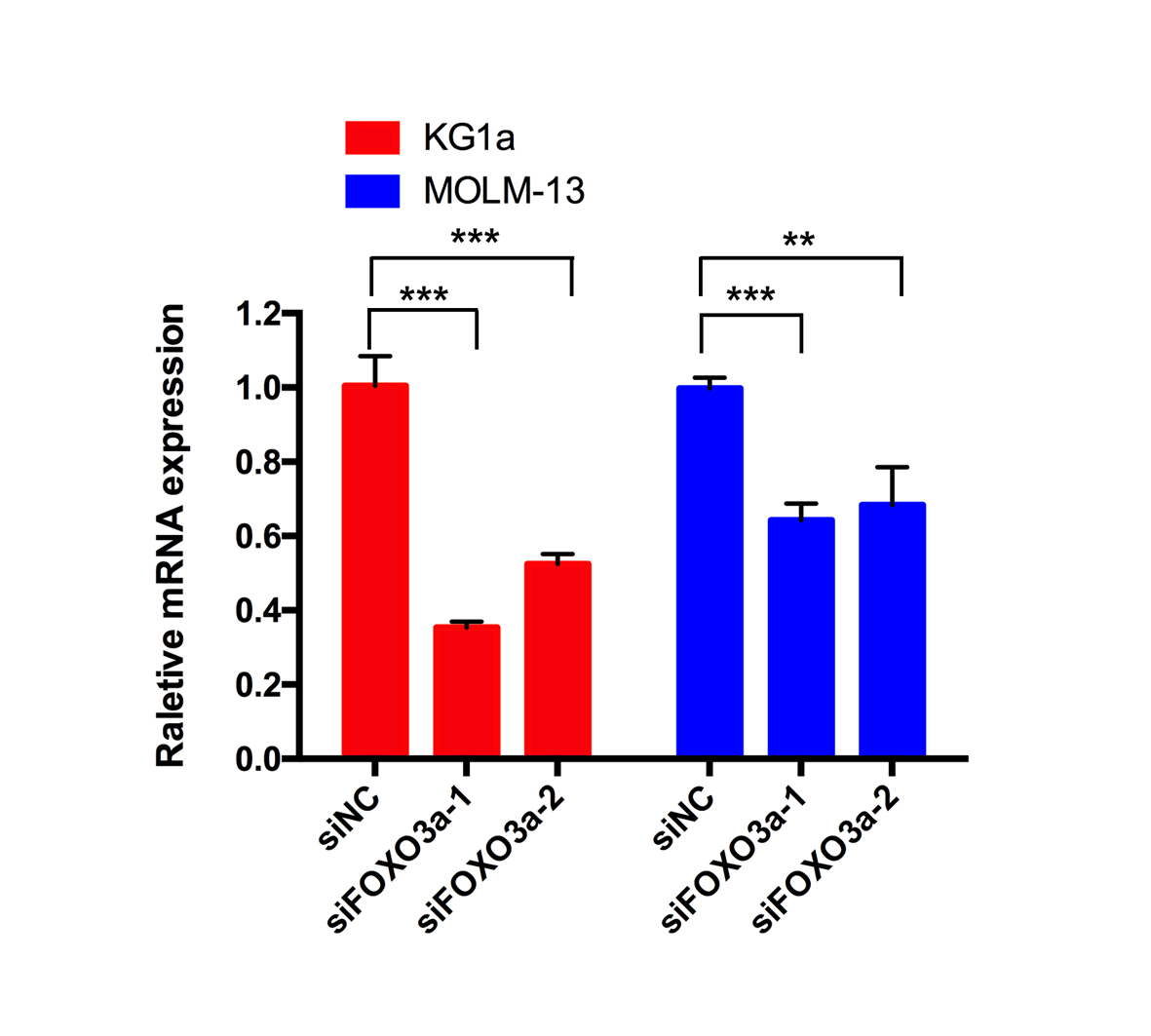
**

**Supplementary Fig. 4 FOXO3a Phosphorylation on T^32^ was unchanged in WTIP-overexpressing KG1a and MOLM-13 cells.** KG1a and MOLM-13 cells transfected with WTIP or control vectors were treated with doxycycline, FOXO3a phosphorylation on T^32^ was analyzed by western blot.

**
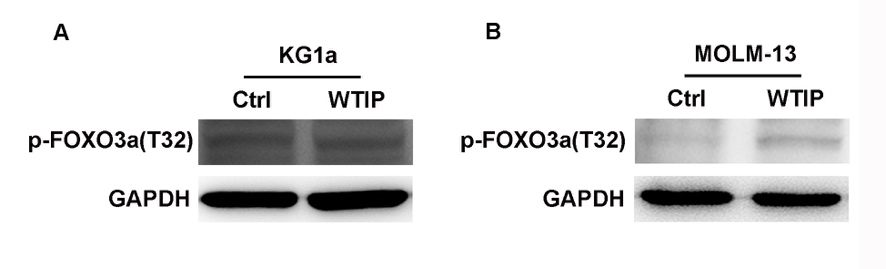
**
